# Screening a Broad Range of Solid and Haematological Tumour Types for CD70 Expression Using a Uniform IHC Methodology as Potential Patient Stratification Method

**DOI:** 10.3390/cancers11101611

**Published:** 2019-10-22

**Authors:** Tal Flieswasser, Valérie Camara-Clayette, Alina Danu, Jacques Bosq, Vincent Ribrag, Piotr Zabrocki, Luc Van Rompaey, Hans de Haard, Karen Zwaenepoel, Evelien Smits, Patrick Pauwels, Julie Jacobs

**Affiliations:** 1 Center for Oncological Research (CORE), University of Antwerp, 2610 Wilrijk, Belgium; tal.flieswasser@uantwerpen.be (T.F.); karen.zwaenepoel@uza.be (K.Z.); evelien.smits@uza.be (E.S.); patrick.pauwels@uza.be (P.P.); julie.jacobs@uantwerpen.be (J.J.); 2 Department of Pathology, Antwerp University Hospital, 2650 Edegem, Belgium; 3 Translational Hematology Unit, AMMICa INSERMUS23/CNRS UMR3655, Gustave Roussy Cancer Campus, 94805 Villejuif, France; valerie.camara-clayette@gustaveroussy.fr; 4 Hematology Department, Gustave Roussy Cancer Campus, 94805 Villejuif, France; Alina.danu@gustaveroussy.fr; 5 Pathology Laboratory, Gustave Roussy Cancer Campus, 94805 Villejuif, France; jacques.bosq@gustaveroussy.fr; 6 Département des Innovations Thérapeutiques et Essais Précoces (DITEP), Université Paris-Saclay, Gustave Roussy Cancer Campus, 94805 Villejuif, France; vincent.ribrag@gustaveroussy.fr; 7 INSERM U1170, 94805 Villejuif, France; 8 Argenx, 9052 Zwijnaarde, Belgium; pzabrocki@argenx.com (P.Z.); lvanrompaey@argenx.com (L.V.R.); 9 Center for Cell Therapy and Regenerative Medicine, Antwerp University Hospital, 2650 Edegem, Belgium

**Keywords:** CD70, CD27, immunohistochemistry, biomarker, immune checkpoint

## Abstract

The constitutive expression of CD70 has been described in various haematological and solid tumour types. In addition, the co-expression of its receptor in tumours has been demonstrated, mediating tumour cell proliferation. Although CD70 expression is a prerequisite to enrol patients in solid tumour clinical trials using anti-CD70 immunotherapy, there is currently no standardised test to evaluate CD70 expression. These differences in immunohistochemistry (IHC) protocols make it challenging to compare the expression levels that were obtained in different studies, pointing out the need for one uniform methodology. In this retrospective study, over 600 tumour samples from different solid and haematological malignancies were analysed while using one validated IHC method. CD70 and CD27 expression was demonstrated in a broad range of tumour types. In solid tumours, 43% demonstrated CD70 positivity with the highest degree in renal cell carcinoma (79.5%). Kaposi sarcoma showed no CD70 expression on the tumour cells. In lymphoma samples, 58% demonstrated CD70 positivity. Moreover, the co-expression of CD70 and CD27 was observed in 39% of lymphoma samples. These findings highlight the need to further explore anti-CD70 therapies in a broad range of CD70 expressing tumour types and in doing so, implementing one standardised protocol to define CD70 overexpression to use it as a diagnostic tool.

## 1. Introduction

Numerous successful clinical trials have led to a great number of FDA approvals for PD-1/PD-L1 blockade therapy in a spectrum of different tumour types: melanoma, non-small cell lung cancer, urothelial carcinoma, classic Hodgkin lymphoma, renal cell carcinoma, Merkel cell carcinoma, hepatocellular carcinoma, gastric carcinomas, and head and neck squamous cell carcinoma. Although the blockage of the PD-1/PD-L1 axis has elicited durable clinical responses in many patients, only 10–40% show clinical response to monotherapy, pointing out the possibility that alternative pathways may have significant impact on the antitumor immune activity in patients with various tumour types [1,2]. 

In this regard, CD70 is a ligand of the tumour necrosis factor superfamily, of which its expression is tightly regulated under physiological conditions: it is only transiently expressed on antigen-activated T and B cells, mature dendritic cells, and a unique population of antigen presenting cells, which is exclusively localised in the gut lamina propria [3,4,5]. Its unique receptor, CD27, acts in a costimulatory pathway, leading to proliferation, differentiation, and survival signals. Depending on the amount, duration, and timing of CD70 expression, CD27 signalling might either lead to improved T cell function or T cell dysfunction [3,6].

The overexpression of CD70 on tumour cells has been described in multiple haematological malignancies and carcinomas [7,8,9]. This overexpression facilitates immune suppression through CD27 signalling in the tumour microenvironment by enhanced survival of regulatory T cells, induction of T cell apoptosis, and skewing T cells towards a T cell exhausted phenotype [10,11,12,13]. In addition, many of the haematological malignancies also co-express CD27 on the tumour cells, which enhances tumour cell proliferation and survival [14,15]. For myeloid malignancies, CD70 was demonstrated to play a critical role in leukemic stem cell maintenance and proliferation [14,16]. Consequently, anti-CD70 immunotherapy is being evaluated in clinical trials and it has been associated with objective clinical responses against various CD70 positive malignancies [17]. Thereof, 92% response rates could be seen in acute myeloid leukaemia patients while using CD70-targeting antibodies in a Phase 1 trial [18].

Different methods to detect CD70 expression in tissues have been described, such as real-time PCR and western blot [19]. To date, immunohistochemistry (IHC) remains the most frequently used method for determining CD70 expression due to its simple and rapid detection of protein expression levels and distribution. Although CD70 positivity of the tumour cells is a prerequisite to enter solid tumour clinical trials while using anti-CD70 immunotherapy, there is currently no uniform IHC assay for evaluating CD70 expression. Consequently, differences in the use of antibody clones and in thresholds for positivity lead to discrepancies in the percentage of CD70 positive cases within identical tumour types, pointing out the need for one uniform and validated methodology [20]. In this retrospective study, we assessed the degree of CD70/CD27 expression across various tumour types while using one standardised and validated IHC assay.

## 2. Results

### 2.1. CD70 Staining Patterns in Solid Tumour Types

**Figure 1:**
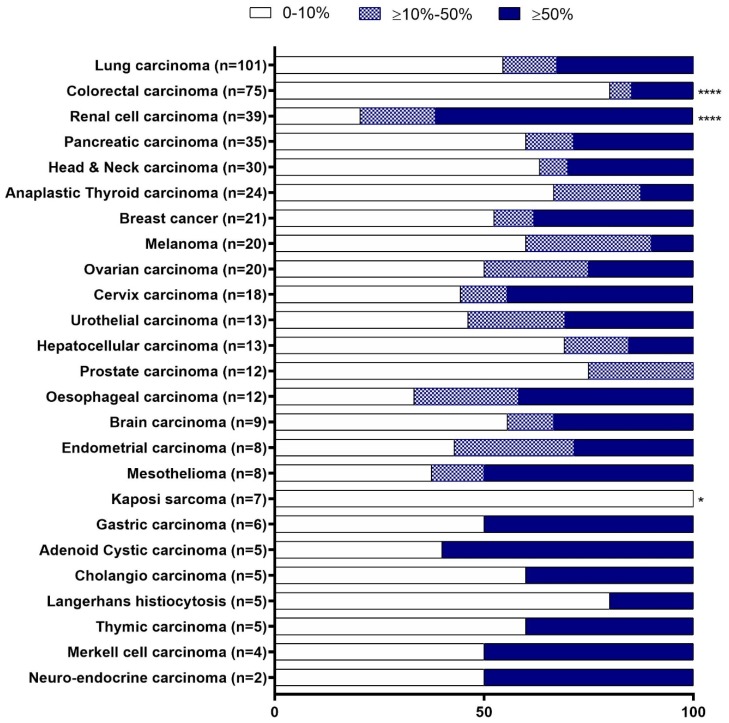
Proportion of CD70 staining among solid tumour types. CD70 staining was categorised in 3 different groups based on the percentage of tumour cells staining positive: expression levels ranging from 0 to 10% (empty bars); between 10 to 50% (patterned bars); and, expression levels above 50% (solid bars). * *p* < 0.05, **** *p* < 0.0001. For statistics, tumour types with *N* < 5 were not considered in the statistical analysis.

**Figure 2:**
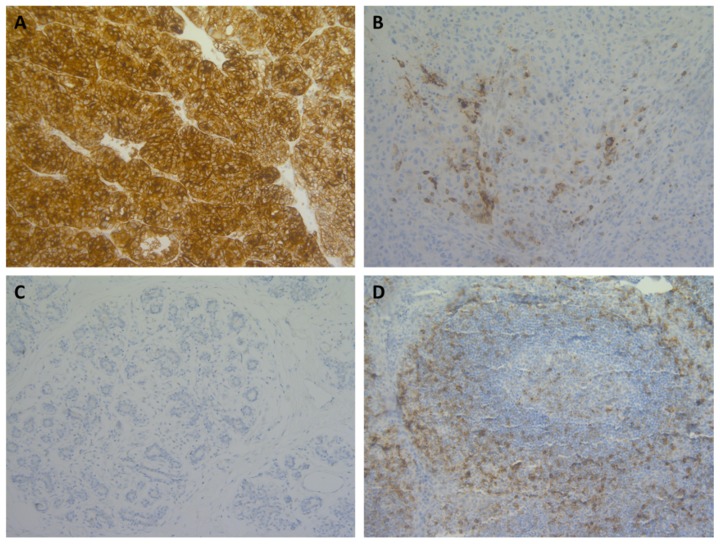
Micrographs of CD70 immunohistochemical stainings across various solid tumour types showing range of staining percentages. (**A**) >50% CD70 staining on tumour cells in renal cell carcinoma; (**B**) 10–50% staining in melanoma; (**C**) <10% staining in breast cancer; and, (**D**) CD70 staining in control tissue (tonsil). Magnitude 200×.

CD70 expression was assessed in 25 different solid tumour types (*N* = 496), which ranged from two to 101 samples per tumour type (Figure 1). The staining of CD70 in various tumour types was localised either on the membrane or in the cytoplasm. Intensity of CD70 expression was categorised as no, weak, moderate, or strong staining (Figure 2).

CD70 expression was divided into three groups; no/low (0–10%), moderate (11–50%) and strong expression (>50%), based on the percentage of CD70 positive cells (of any intensity) (Figure 1). The cut-off value of 10% was used to determine CD70 positivity, which is in accordance with the cut-off used in clinical trials of anti-CD70 therapy [17]. CD70 positivity (>10%) was observed in all solid tumour types, with the exception of Kaposi sarcoma (*N* = 7) (Figure 1). CD70 positivity was significantly most prevalent in the samples of renal cell carcinoma (79.5%), as compared to all other tumour types (*p* < 0.0001). In addition, the majority of samples from oesophageal carcinoma (66.7%, *N* = 12) and mesothelioma (62.5%, *N* = 8) showed CD70 positivity. CD70 expression in more than 50% of the tumour cells was mainly observed in renal cell carcinoma (61.5%, *N* = 39), gastric carcinoma (50%, *N* = 6), and adenoid cystic carcinoma (60%, *N* = 5). Contrarily, CD70 negative tumour cells (0–10%) were mostly observed in prostate carcinoma (75%, *N* = 12), Langerhans cell histiocytosis (80%, *N* = 5), Kaposi sarcoma (100%, *N* = 7), and colorectal carcinoma (80%, *N* = 75), whereby a significant lower expression of CD70 was observed in the latter two groups as compared to all other tumour types (*p* = 0.012 and *p* < 0.0001, respectively). 

Specimens of primary and metastatic biopsies were obtained for most tumour types, with the exception of Kaposi sarcoma, thymic, gastric, cholangio, and thyroid carcinoma. CD70 expression from both primary and metastatic tumour biopsies showed similar percentages of CD70 positive samples in both resection specimens for most tumour types, although it should be noted that, for certain tumour types, the amount of samples per subgroup (primary versus metastasis) was limited. In several tumour types, CD70 positivity was higher in metastatic specimens as compared to primary tissue biopsies, such as lung carcinoma (85.7%, *N* = 7 vs 40%, *N* = 80) (*p* = 0.048) and pancreatic carcinoma (87.5%, *N* = 8 vs 26.1%, *N* = 23) (*p* = 0.004). 

We observed different CD70 staining intensities amongst the tumour types. Ovarian carcinoma and endometrial carcinoma mainly showed weak positive staining. Moderate staining was mostly observed in mesothelioma, while most patients with renal cell carcinoma exhibited strong staining when compared to other solid tumour types (Supplementary Figure S1).

### 2.2. CD70 and CD27 Protein Expression in Haematological Malignancies

**Figure 3:**
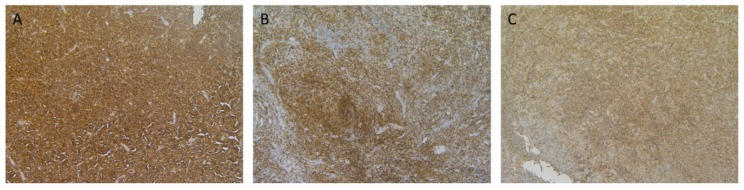
Representative micrographs of CD70 immunohistochemical staining on samples of patients with haematological malignancies. (**A**) CD70 staining in a mantle cell lymphoma; (**B**) cutaneous T-cell lymphoma; and, (**C**) diffuse large B cell lymphoma is shown. Magnitude 100×.

Lymphoma samples (*N* = 130) were analysed for CD70 and CD27 expression and they were categorised in five histological subgroups according to their defined pathology: mantle cell lymphoma (MCL, *N* = 65), peripheral T cell lymphoma (PTCL, *N* = 50), NK/T-cell lymphoma (NKTCL, *N* = 4), anaplastic large cell lymphoma (ALCL, *N* = 3), and diffuse large B cell lymphoma (DLBCL, *N* = 8). Lymphoma samples displayed different CD70 expression levels, as shown in Figure 3. Of the 50 PTCL samples, four had unknown CD70 levels due to tissue that was not representative. Of the 50 PTCL, three ALCL and eight DLBCL samples, seven, one, and three patient(s) had unknown CD27 levels, respectively.

**Figure 4:**
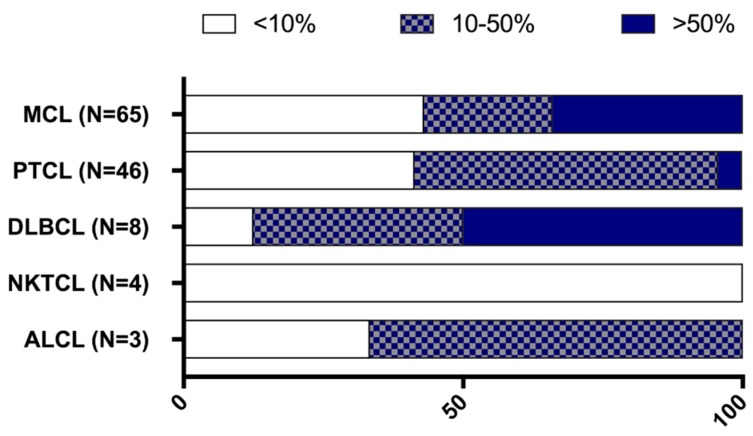
Proportion of CD70 staining among various lymphoma subsets. CD70 staining was categorised in three different groups: CD70 positive tumour cells ranging from 0 to 10% (white bars); between 10% to 50% (patterned bars); and expression levels above 50% (blue bars). ALCL, anaplastic large cell lymphoma; DLBCL, diffuse large B cell lymphoma; MCL, mantle cell lymphoma; NKTCL, NK/T cell lymphoma; PTCL, peripheral T-cell lymphoma.

CD70 positive tumour cells (>10%) were observed in all lymphoma types, with the exception of NKTCL patients’ samples, which did not show any expression of CD70 (Figure 4). CD70 expression was detectable in 59% of PTCL. Twenty-five patients with PTCL (54%) had CD70 expression levels between 10–50% and two patients (4%) had very high levels of CD70 (>50%). In addition, seven out of eight DLBCL samples (88%) and two out of three ALCL samples showed CD70 positivity. 

A larger cohort of 65 MCL patients’ samples were analysed for CD70 expression. An overview of the patient characteristics can be found in Supplementary Table S1. Thirty-seven tumour samples (57%) were collected before treatment and 28 (43%) at relapse. 52 samples (80%) were from patients at stage III or IV of the disease. Most of the samples were collected from lymph nodes (57%) and gastrointestinal tract (18%). CD70 positivity was found in 37 MCL samples (57%), of which 26 samples (70%) displayed CD70 expression on >50% of the tumour cells (Figure 4, Table 1). We found no significant difference in CD70 expression between the samples at diagnosis or relapse. Although a slight enrichment in CD70 expression in lymph nodes could be found in the samples of relapsed patients as opposed to the samples that were taken at diagnosis (56% vs 43%, respectively) (P>0.05) (Table 1). 

**Figure 5:**
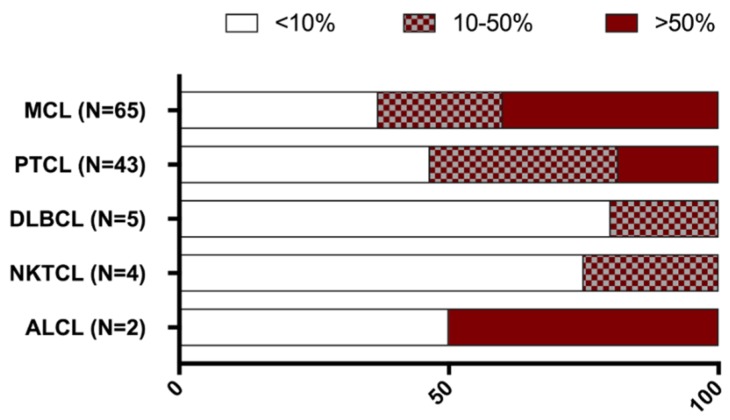
Proportion of CD27 staining among various lymphoma subsets. CD27 staining was categorised in three different groups: CD27 positive tumour cells ranging from 0 to 10% (white bars); between 10 to 50% (patterned bars); and expression levels above 50% (red bars). ALCL, anaplastic large cell lymphoma; DLBCL, diffuse large B cell lymphoma; MCL, mantle cell lymphoma; NKTCL, NK/T cell lymphoma; PTCL, peripheral T-cell lymphoma.

CD27 expression was assessed in all lymphoma samples (Figure 5). All of the subsets showed CD27 positivity (>10% CD27 positive tumour cells) (Figure 5). CD27 positivity was observed in 23 patients with PTCL (54%), one out of two ALCL patients, one out of four patients with NKTCL, and one out of five patients with DLBCL. 

**Figure 6:**
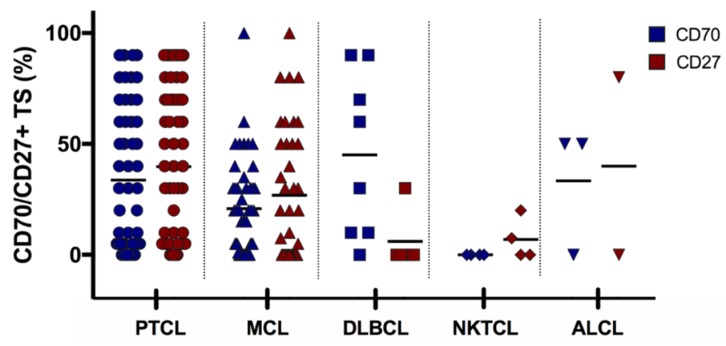
Overview of CD70 and CD27 expression in 117 lymphoma samples. Each symbol represents a different patient’s sample and bars represent the mean. ALCL, anaplastic large cell lymphoma; DLBCL, diffuse large B cell lymphoma; MCL, mantle cell lymphoma; NKTCL, NK/T cell lymphoma PTCL, peripheral T-cell lymphoma. TS, tumour sample

For MCL, CD27 expression was detectable in 41 cases (63%), 24 at diagnosis (65%), and a slight drop in the expression at relapse was observed (17 samples (61%), *p* > 0.05). Co-expression of CD70 and CD27 was observed in 19 PTCL (46%, *N* = 41), one ALCL (*N* = 2), and one DLBCL (*N* = 5) patient(s) (Figure 6).

**Figure 7:**
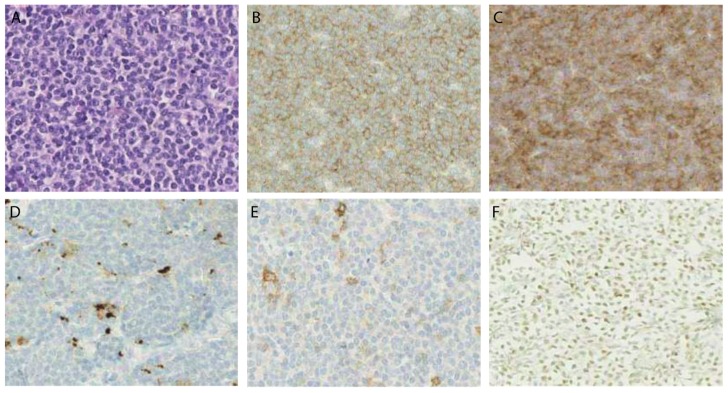
Micrographs of immunohistochemical stainings in one MCL patient sample. Sequential cuts of one MCL patient sample showing staining of (**A**) HE; (**B**) CD70; (**C)** CD27; (**D)** PD-L1; (**E)** PD-1; and, (**F)** nuclear staining of MCL cells for cyclin D1. Magnitude 40×.

Twenty-five MCL samples (38%) were positive for both CD70 and CD27, whereof 60% (15 samples) showed double positivity within ≥ 50% of tumour cells. The majority of double positive samples (60%) was collected from patients at diagnosis (*p* > 0.05). Twenty-eight MCL tumour samples (43%) had a very low CD70 expression (< 10%). Thereof, 12 samples also displayed a very low level of CD27 (Table 1). Figure 7 shows representative images of CD70 and CD27 expression in MCL samples. Cyclin D1 (Figure 7F), CD3, and CD20 stainings (Supplementary Figure S2) were used as control markers to ensure specific CD70/CD27 staining on the malignant cells.

The co-expression of CD70 and CD27 in all haematological samples showed a weak positive correlation (*p* = 0.002, correlation coefficient (R) = 0.3). 

### 2.3. Programmed Cell Death Protein 1 (PD-1) and Its Ligand (PD-L1) Expression in MCL Patient Cohort 

In addition to CD70/CD27 staining, the expression of PD-L1 and PD-1 was analysed in the large MCL cohort to estimate the expression of other relevant immunotherapeutic targets (Figure 7D,E). PD-L1 was undetectable in the majority of MCL samples. Twelve tumour samples (18%) had between 5–25% PD-L1 positive cells and they were considered as PD-L1 positive (PD-L1+) (Table 2). Seven PD-L1-positive samples (58%) were newly diagnosed MCL patients (19% of all samples at diagnosis) and 5 (42%) relapsed/refractory MCL patients (18% of all relapse samples).

**Figure 8:**
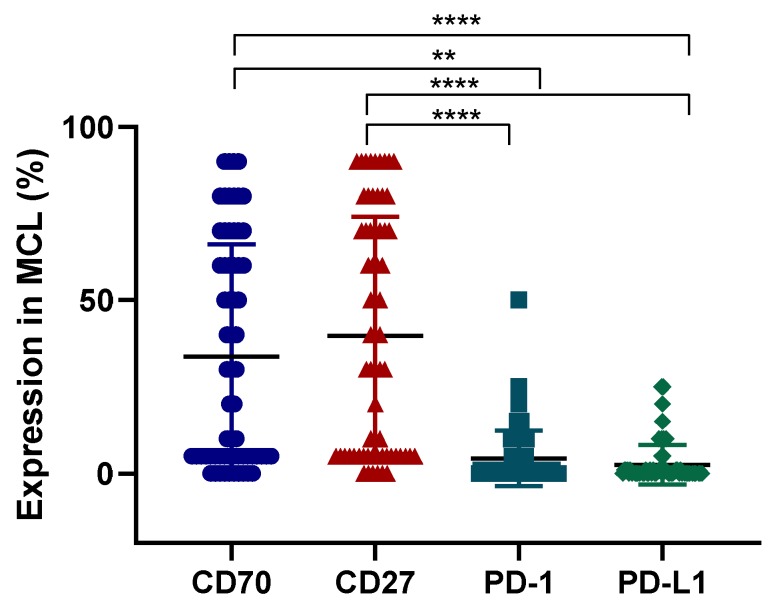
Expression levels of CD70, CD27, PD-1, and PD-L1 in MCL cohort. The expression of CD70 CD27, PD-1, and PD-L1 in a total of 65 MCL samples. Y axis shows percentage of positive cells for a particular protein. Each symbol represents a different patient. ** *p* < 0.01, **** *p* < 0.0001.

In total, 23 samples (35%) were positive for PD-1 with staining typically between 5–25% of cells per sample. Thirty-eight samples (58%) were double negative for PD-L1 or PD-1 (22 at diagnosis and 16 at relapse) and only eight samples (12%) were double positive for both PD-L1 and PD-1. A weak positive correlation was observed between PD-L1 and PD-1 with (*p* = 0.05, *R* = 0.34). Interestingly, significantly higher expression levels of CD70 and CD27 could be found as opposed to PD-L1 and PD-1 (*p* < 0.0001) (Figure 8). 

## 3. Discussion

Our study demonstrated CD70 expression with varying intensities in all tumour types, excluding Kaposi sarcoma and NKTCL. Of note, for some tumour types, the number of samples was too limited to significantly determine CD70 positivity and predict the benefit from anti-CD70 therapy.

In addition, we only had limited data regarding the clinicopathological parameters of patients, which did not allow us to analyse links between CD70 expression and additional factors, such as previous treatments effects on CD70 expression or disease stage. Nevertheless, we were able to identify solid tumours and lymphomas with different CD70 expression levels and we believe that our unified method can improve the stratification of patients for immunotherapy.

CD70 overexpression on tumour cells is associated with poor prognosis [21,22,23]. Co-expression of CD27 has also been described in several tumours of hematopoietic lineage, implicating a possible role for the CD70-CD27 axis in the regulation of tumour cell proliferation and survival [24,25]. CD27 expression is almost exclusively restricted to immune cells in solid tumour types, in contrast to CD70-CD27 co-expression on tumour cells in hematopoietic malignancies [20]. Nonetheless, persistent signalling of CD27 can occur in the tumour micro-environment through its expression on tumour-infiltrating lymphocytes (TILs), particularly on immune suppressive Tregs [26]. Therefore, CD70-CD27 signalling is one of the immune escape mechanisms that takes place in the tumour microenvironment and it facilitates the evasion of cancer cells from the host’s immune system [10,27]. 

Targeting the CD70/CD27 pathway has emerged as a potential novel immunotherapeutic strategy, which is already being explored in several early phase clinical trials testing CD70- and CD27-targeting antibodies in various malignancies [17,28,29], but, to date, no standardised method allowing for a comparison of CD70 expression in different tumour types has been published.

The detection of patients’ CD70 expression status should be performed in a consistent manner to correctly stratify and monitor patients for anti-CD70 immunotherapy: both the technical staining procedure as well as the interpretation of the test must be reproducible. Although IHC remains the most frequently used method to determine CD70 expression, dissimilarities are seen in the percentage of CD70 positive cases within identical tumour types due to e.g., different antibodies or cut-off values (summarised in Jacobs et al. [20]). These discrepancies highlight the need for one uniform and validated methodology for stratifying tumour types for anti-CD70 immunotherapy. In this study, no equipment for computer-assisted image classification was used, since this is not yet available in the majority of clinical pathology laboratories. Therefore, positivity was assessed by visual annotation of pathologists, to closely resemble clinical practice. Rizzardi et al. have already demonstrated the strong similarities between computer-based and visual annotation, ensuring the reproducibility of our results with computer-assisted image classification in the future [30].

The variation in cut-off values to define CD70 expression also leads to inconsistent interpretations regarding CD70 expression profiles within identical tumour types. In our study, we compared CD70 expression in 496 solid tumour specimens while using one uniform CD70 IHC method, which allowed for us to compare CD70 expression within one specific neoplasm type as well as among different tumour types. 

The highest number of CD70 positive cells was detected in renal cell carcinoma. CD70 expression in renal cell carcinoma has already been described in several studies, although with varying positivity for CD70. While Law CL et al. [31] reported CD70 expression in 30% to 68% of clear cell and 40% of papillary renal cell carcinoma (cut-off value of 25%), Junker K. et al. [32] showed that all clear cell samples (41 in total) and only one papillary sample (19 in total) were positive for CD70 (cut-off unknown). Our study demonstrated that 79.5% of all renal cell carcinoma patients (39 in total) were positive for CD70. In the case of brain carcinoma, samples were examined for CD70 expression, whereby Ryan MC et al. [33] demonstrated CD70 positivity in 10% of the samples (59 in total) using 1% as cut-off. Contrarily, Wischhusen J et al. [12] found that 42% of glioblastoma patients (12 in total) were positive for CD70 (cut-off unknown). Similarly, our study showed that 44.4% of all brain carcinoma patients (nine in total) were CD70 positive. Surprisingly, while Ryan MC et al. [33] reported that only 2% of breast cancer patients were positive for CD70 (*N* = 204) while using a cut-off of 1%, our study showed that 47.6% breast cancer patients were CD70 positive (cut-off of 10%).

We tested both primary and metastatic solid tumour specimens to determine CD70 expression. In tumour types, such as renal cell carcinoma, hepatocellular carcinoma, and head & neck carcinoma, tissues from both primary and metastatic sites were similarly positive for CD70. Similar findings of the matched primary and metastatic tissue samples of patients with renal cell carcinoma were observed by Law CL et al. [31] and Adam PJ et al. [34]. Interestingly, we showed that the number of CD70 positive cells appeared to be even higher in metastatic tissue as compared to primary specimens in pancreatic carcinoma and lung carcinoma. Although we acknowledge that, for certain tumour types, a limited number of samples were available and thus more samples would be needed to draw more precise conclusions.

Furthermore, we also determined CD70 expression in 130 lymphoma samples and detected CD70 positive cells in all tested lymphoma subtypes, except for the NKTCL samples. Though it should be noted that a limited number of samples of NKTCL were analysed. On the contrary, Yoshino et al. [35] showed that CD70 was expressed in 33% of nasal NKTCL tissues (total of 21 Japanese patients), although their cut-off value was not stated. 

Interestingly, more than half of MCL patients appeared to be CD70 positive in our study. To date, immune checkpoint inhibitors, such as PD-1 and PD-L1, are mostly used with promising results in different types of cancer, although limited data are available on immune checkpoint inhibitors in MCL. Based on the few available studies, PDL-1/PD-1 expression is usually not detectable by IHC analysis on MCL cells [36,37,38]. Nevertheless, using a cut-off of 5%, we could detect small numbers of PDL-1/PDL1 positive cells in 18% and 35% of MCL samples, respectively, and much lower numbers of double positive samples were observed. Furthermore, in a phase I study with nivolumab, four patients with MCL were treated, but none of the MCL patients achieved an objective response in this study [39]. This underscores the need to search for alternative approaches to efficiently target MCL. In this regard, our study demonstrated the expression of CD70 and CD27 in the majority of MCL samples and co-expression in 38% of patients, suggesting that the CD70/CD27 axis is a more attractive target for therapy in case of MCL patients. 

Therefore, targeting the CD70/CD27 axis could be of great relevance in treating cancer types that currently lack effective treatment strategies. In this regard, a breakthrough was recently achieved for cusatuzumab (ARGX-110), an anti-CD70 targeting antibody, in combination with Azacitidine in a Phase 1/2 trial in acute myeloid leukaemia (AML). The response rates were observed in 11 out of 12 AML patients, including 10 complete remissions, and median response duration was 6.9 months as of the data cut-off date on July 16, 2018 [18]. As we demonstrate high expression levels of CD70/CD27 in other tumour types using our uniform IHC method, these results underline the potential of anti-CD70 immunotherapy not only in AML, but also in other haematological malignancies and solid tumour types where CD70 is overexpressed. 

## 4. Materials and Methods 

### 4.1. Patient Selection and Tissue Specimen

620 formalin fixed paraffin embedded (FFPE) specimens were collected from different tumour types, of which 496 samples from solid tumours (Figure 1) and 130 samples from haematological malignancies (Figure 6). The Ethics committee of Antwerp University Hospital and the Ethics committee of Institute Gustave Roussy approved this study (EC13/47/469).

### 4.2. CD70 IHC

5 µm-thick sections were exposed to heat-induced antigen retrieval by incubation in a high pH buffer for 20 min. at 97 °C (PT-Link) (DAKO, Glostrup, Denmark). Subsequently, endogenous peroxidase activity was quenched by incubating the slides in peroxidase blocking buffer (DAK0) for 5 min. CD70 IHC was performed as previously described [26]. Shortly, anti-CD70 antibody (R&D systems, Minneapolis, MN, Canada; Clone 301731, 1:40) was incubated at room temperature for 20 min., followed by mouse enhanced polymer-based linker (30 min., DAKO) and visualised via a DAKO autostainer Link 48 instrument while using the Envision FLEX+ detection kit (DAKO) according to the instructions of the manufacturer. Tonsil tissue was included in each staining run and used as positive and negative tissue control (Figure 2D). Furthermore, biopsies were checked for internal positive control (e.g., lymphocytes) and negative control (e.g., epithelial cells, muscular layer, neuronal cells). Two independent observers as well as one pathologist performed scoring. Validation of the most optimal immunohistochemical CD70 staining protocol was performed at the University Hospital of Antwerp (UZA) by manual analysis. FFPE material of human spleen and tonsil, renal cell carcinoma, as well as cell line material in agar block with known high (MJ), intermediate (MM1-S), and low (SU-DHL-6) CD70 copy number were used for the comparison of multiple commercial antibodies available for CD70 staining. Based on validation parameters repeatability (intra-run precision), reproducibility (inter-run precision), accuracy, specificity (using normal tissue and cancer-related tissue), and sensitivity (using agar cell block of cell lines with different levels of CD70 expression), CD27Ligand, clone 301731 (R&D Systems) showed the most specific staining. Further information on the evaluation criteria can be found in supplementary methods.

### 4.3. CD27, PD-1 and PD-L1

CD27, PD-1 and PD-L1 stainings were performed on a BenchMark ULTRA Ventana (Roche Diagnostics, Indianapolis, IN, USA). For CD27, the samples were pre-incubated in Ultra CC1 for 64 minutes, incubated with anti-CD27 monoclonal antibodies for 92 min. at 37 °C (Thermo Scientific, Rockford, IL, USA; Clone 137B4, 1:20), and detected by the ultraView plus detection kit and Amplification kit (Roche diagnostics). 

For PD-1 and PD-L1 detection, the samples were pre-incubated in CC1 solution (Roche Diagnostics) for 32 min. and 64 min. in case of PD-1 and PD-L1 detection, respectively, and then incubated with anti-PD-1 (Abcam, Cambridge, UK, clone NAT105, 60 min., RT) and anti-PD-L1 (Cell Signaling Technology, Danvers, MA, USA, Clone E1L3N®, 60 min., 37 °C). Dilutions of primary antibodies were adjusted, depending on the type of fixator used (in case of PD-1 1:750 for formal solution and 1:350 for AFA solution; in case of PD-L1 1:2000 for formal solution and 1:1000 for AFA solution). Control tissue was used to determine the correct antibody dilutions for each fixator. For detection, OptiView kit was used and, in addition, OptiView Amplification kit was used in the case of PD-L1 detection.

### 4.4. Statistical Analysis

Differences in expression levels of CD70 were assessed by Mann–Whitney *U* tests. In each comparison, one tumour type was compared to all others. Positivity (primary, metastasis, diagnosis, relapse) was assessed by Chi-square or Fisher’s Exact test. Correlations between CD70, CD27, PD-1, and PD-L1 were investigated by Pearson correlation coefficients and McNemar tests. Differences in the expression levels of these four markers were studies by Wilcoxon Signed Rank tests. All analyses were performed using SPSS version 25 and significance was reached if *p* < 0.05.

## 5. Conclusions

We have shown strong CD70 overexpression in different solid and haematological malignancies. Moreover, we could demonstrate higher relevance of the CD70/CD27 axis for potential therapeutic application in MCL in comparison to the PD-L1/PD-1 axis. These findings highlight the potential of anti-CD70 therapies in a broad range of tumour types and, in doing so, implementing one standardised protocol to define CD70 overexpression to use it as a diagnostic tool.

## Figures and Tables

**Table 1:** CD70 and CD27 expression analysis in mantle cell lymphoma (MCL) samples.

CD70/CD27	Diagnosis *N* = 37	Relapse *N* = 28	All Tumour Samples *N* = 65
CD70^+^, *n* (%)	20 (54)	17 (61)	37 (57)
Staining %, median (range)	60% (10–90%)	60% (10–90%)	60% (10–90%)
≥ 50% staining, *n* (%)	13 (65)	13 (76)	26 (70)
CD27^+^, *n* (%)	24 (65)	17 (61)	41(63)
Staining %, median (range)	60% (30–90%)	70% (10–90%)	70% (10–90%)
≥ 50% staining, *n* (%)	16 (67)	13 (76)	29 (71)
CD70^+^, CD27^+^, *n* (%)	15 (41)	10 (36)	25 (38)

**Table 2:** PD-1 and PD-L1 expression analysis in MCL samples.

PD-L1/PD-1	Diagnosis *N* = 37	Relapse *N* = 28	All Tumour Samples *N* = 65
PD-L1^+^, *n* (%)	7 (19)	5 (18)	12 (18)
Staining %, median (range)	10% (10–25%)	10% (5–25%)	10% (5–25%)
PD-1^+^, *n* (%)	14 (38)	9 (32)	23 (35)
Staining %, median (range)	10% (5–25%)	10% (5–50%)	10% (5–50%)
PD-1^+^, PD-L1^+^, *n* (%)	5 (14)	3 (11)	8 (12)

## Supplementary Materials

The following are available online at https://www.mdpi.com/2072-6694/11/10/1611/s1, Figure S1: Range of CD70 staining intensities in solid tumours, Figure S2: Staining examples in 2 MCL cases, Table S1: Characteristics of MCL patient cohort, Supplementary Methods: Evaluation criteria for selection of CD27Ligand antibody.
