# Supplementary material for: Screening a Broad Range of Solid and Haematological Tumour Types for CD70 Expression Using a Uniform IHC Methodology as Potential Patient Stratification Method
Source: Cancers (Basel). 2019 Oct 22;11(10):1611. doi: 10.3390/cancers11101611 (PMC6826714; doi:10.3390/cancers11101611)
Supplement: Supplementary file 1 [file cancers-11-01611-s001.pdf]

Supplementary Materials: Screening a Broad Range of Solid and Haematological Tumour Types for CD70 Expression Using a Uniform IHC Methodology as Potential Patient Stratification Method

Tal Flieswasser, Valérie Camara-Clayette, Alina Danu, Jacques Bosq, Vincent Ribrag, Piotr Zabrocki, Luc Van Rompaey, Hans de Haard, Karen Zwaenepoel, Evelien Smits, Patrick Pauwels and Julie Jacobs

Table S1. Characteristics of MCL patient cohort ($N = 65$).

Characteristics	*n* (%)
**Sex**	
Male	53 (82)
Female	12 (18)
**Age at diagnosis**	
Median	61
Range	39-94
**ECOG**	
0–1	47 (73)
≥ 2	6 (9)
unknown	12 (18)
**Ann Arbor Staging**	
Stage I–II	11 (17)
Stage III–IV	52 (80)
Unknown	2 (3)
**Metastasis**	
M1	31 (48)
M2	15 (23)
**LDH**	
Normal	35 (54)
Elevated	20 (31)
Unknown	10 (15)
**Biopsy**	
Diagnosis	37 (57)
Relapse	28 (43)
Lymph node	37 (57)
Gastrointestinal tract	12 (18)
Other	16 (25)
**Treatment before biopsy**	
**Rituximab**	
Yes	15 (23)
No	49 (75)
Never Treated	37 (57)
Chemotherapy alone	12 (18)
Unknown	1 (2)
**Aracytine**	
Yes	11 (17)
No	53 (81)
Unknown	1 (2)
**Autologous Stem cell transplantation**	
Yes	7 (11)
No	58 (89)

Supplementary methods

Evaluation criteria for selection of CD27Ligand antibody

CD70 LS/ LifeSpan (LS)-A8811, LS-A8812, LS-A8809 and CD27 Ligand, clone 301731 (R&D systems) were compared for selection of the optimal antibody for CD70 staining. The following evaluation criteria were applied: 1) CD70 IHC expression in spleen and tonsil in agreement with international literature regarding CD70 expression; 2) CD70 IHC expression in cell lines and renal carcinoma cells in agreement with known copy numbers of CD70 present in the cell (determined by flow cytometry); 3) CD70 IHC expression in renal cell carcinoma in agreement with incidence of CD70 positive renal cell carcinoma as described in international literature. Only with the CD27 Ligand, clone 301731 (R&D) a protocol could be generated in which the evaluation criteria were met. Hence, this antibody was selected for further validation. Since CD70 LS-A8809 did not stain the renal carcinoma cells and the staining of the 3 cell lines was not in agreement with the copy numbers of CD70 present in these cells, the specificity of this antibody remains in question. CD70 LS-A8811 showed almost no staining (even after adding a linker) and CD70 LS-A8812 showed problems with specificity since staining was equally strong in epithelial cells and germinal centre.

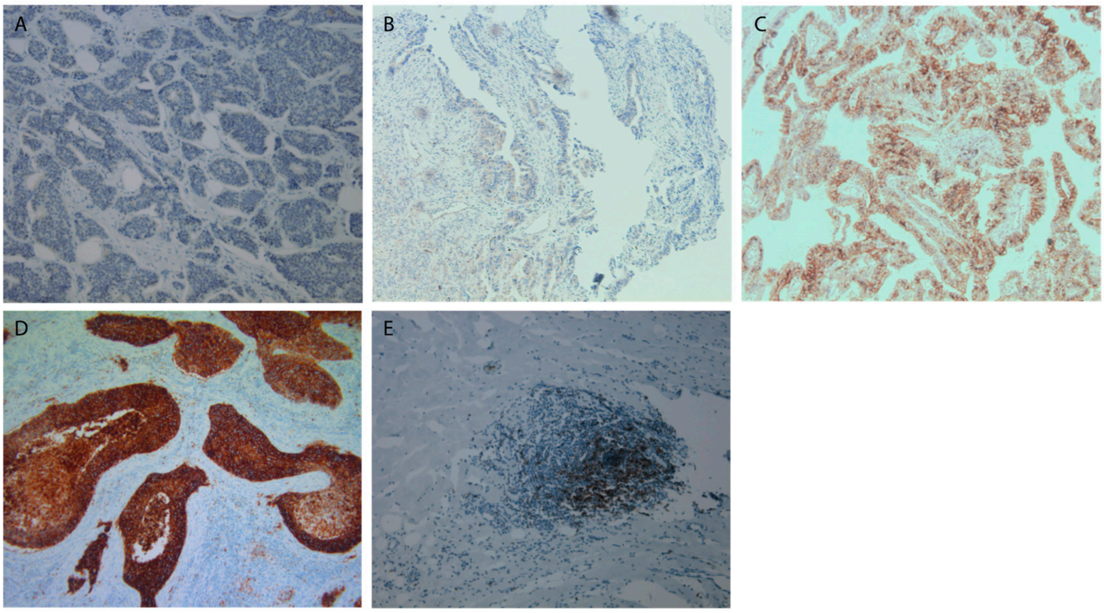

Figure S1. Micrographs of CD70 immunohistochemical staining of tumour tissue of various solid tumour types showing range of staining intensities. (**A**) no CD70 staining; (**B**) weak staining; (**C**) moderate CD70; (**D**) strong CD70 staining; (**E**) CD70 staining in lymphoid aggregates; Magnitude 100×.

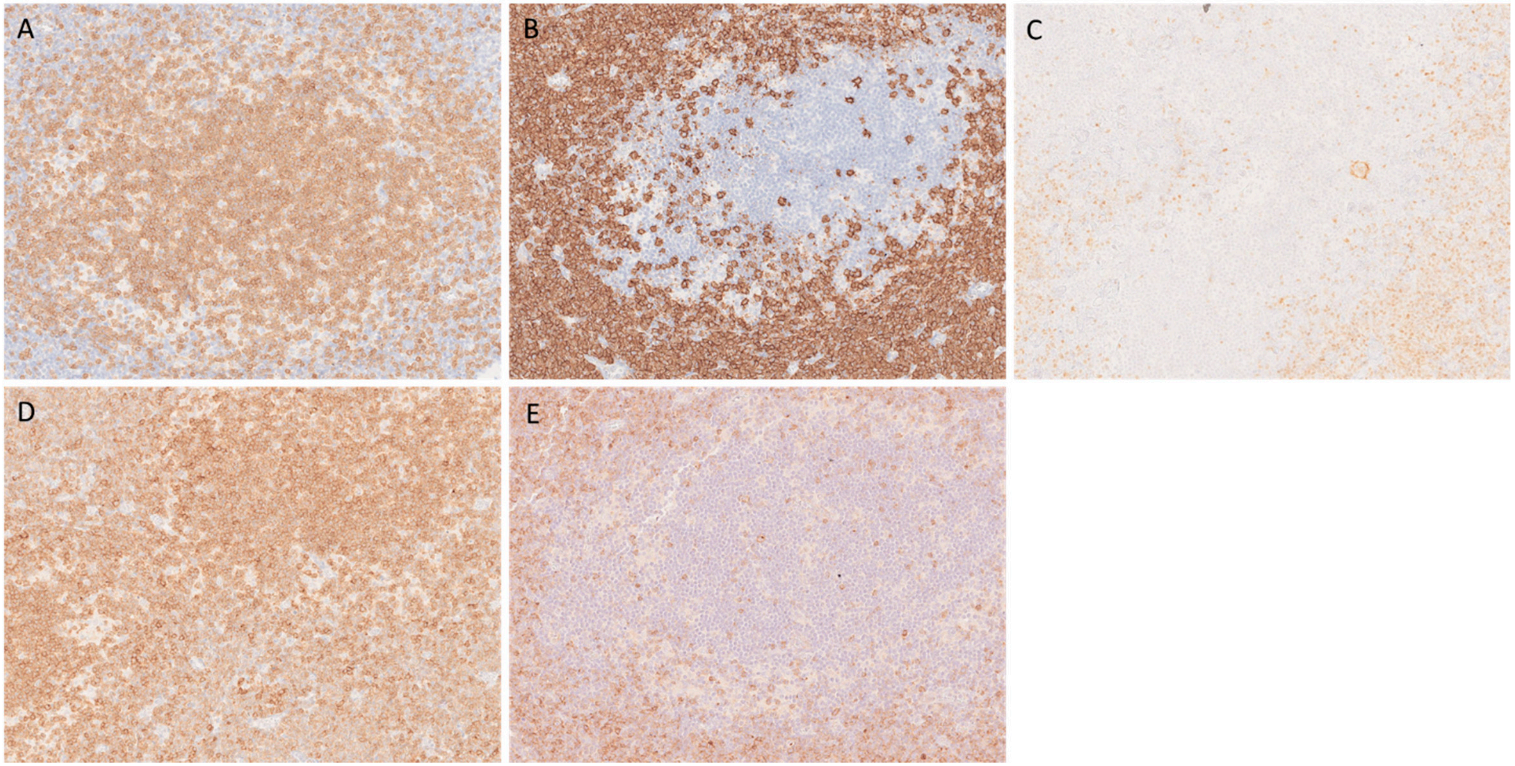

Figure S1. *Case 1.*

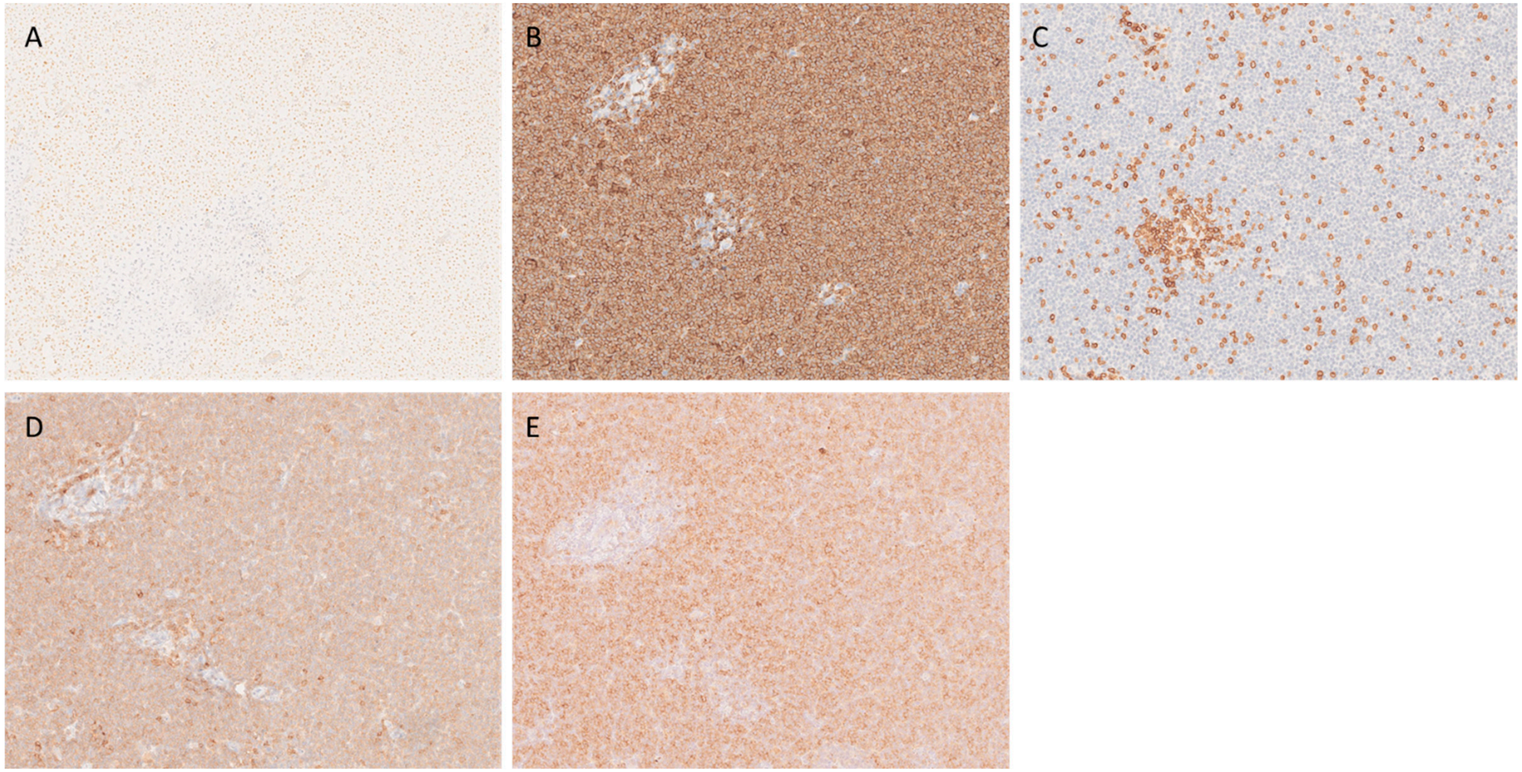

Case 2.

Figure S2. Staining examples in 2 MCL cases. Case 1. Representative example of an MCL sample showing CD20, CD70 and cyclin D1 positive MCL cells and CD3 and CD27 positive nodes; (**A**) CD3 (T lymphocytes); (**B**) CD20; (**C**) cyclin D1 (MCL cells); (**D**) CD27and (**E**) CD70 stainings. Magnitude 10×. Case 2. Representative example of an MCL sample with CD70, CD27, CD20 and cyclin D1 positive tumour cells, and both CD3 and CD27 positive node cells; (**A**) cyclin D1 (MCL cells); (**B**) CD20; (**C**) CD3 (T lymphocytes); (**D**) CD27 and (**E**) CD70 stainings. Magnitude 10×.

© 2019 by the authors. Licensee MDPI, Basel, Switzerland. This article is an open access article distributed under the terms and conditions of the Creative Commons Attribution (CC BY) license (http://creativecommons.org/licenses/by/4.0/).
